# SGLT2-inhibitors effects on the coronary fibrous cap thickness and MACEs in diabetic patients with inducible myocardial ischemia and multi vessels non-obstructive coronary artery stenosis

**DOI:** 10.1186/s12933-023-01814-7

**Published:** 2023-04-01

**Authors:** Celestino Sardu, Maria Consiglia Trotta, Ferdinando Carlo Sasso, Cosimo Sacra, Gerardo Carpinella, Ciro Mauro, Fabio Minicucci, Paolo Calabrò, Michele D’ Amico, Fabrizio D’ Ascenzo, Ovidio De Filippo, Mario Iannaccone, Carmine Pizzi, Giuseppe Paolisso, Raffaele Marfella

**Affiliations:** 1 Department of Advanced Medical and Surgical Sciences, University of Campania “Luigi Vanvitelli”, Piazza Miraglia, 2, 80138 Naples, Italy; 2 Department of Experimental Medicine, University of Campania “Luigi Vanvitelli”, Naples, Italy; 3 Department of Cardiology, Gemelli Molise, Campobasso, Italy; 4 Department of Cardiology, Hospital Cardarelli, Naples, Italy; 5 Department of Cardiology, San Leonardo Hospital, Naples, Italy; 6 Department of Cardio-Thoracic and Respiratory Sciences, University of Campania “Luigi Vanvitelli”, Caserta, Italy; 7 Division of Cardiology, Cardiovascular and Thoracic Department, “Città della Salute e della Scienza”, Turin, Italy; 8 Department of Cardiology, San Giovanni Bosco Hospital, ASL Città di Torino, Turin, Italy; 9 Unit of Cardiology, Department of Experimental, Diagnostic and Specialty Medicine-DIMES, University of Bologna, Bologna, Italy

**Keywords:** SGLT2-I, Mv-NOCS, Inflammatory burden, FCT, MACEs

## Abstract

**Background:**

Sodium–glucose transporter 2 inhibitors (SGLT2-I) could modulate atherosclerotic plaque progression, via down-regulation of inflammatory burden, and lead to reduction of major adverse cardiovascular events (MACEs) in type 2 diabetes mellitus (T2DM) patients with ischemic heart disease (IHD). T2DM patients with multivessel non-obstructive coronary stenosis (Mv-NOCS) have over-inflammation and over-lipids’ plaque accumulation. This could reduce fibrous cap thickness (FCT), favoring plaque rupture and MACEs. Despite this, there is not conclusive data about the effects of SGLT2-I on atherosclerotic plaque phenotype and MACEs in Mv-NOCS patients with T2DM. Thus, in the current study, we evaluated SGLT2-I effects on Mv-NOCS patients with T2DM in terms of FCT increase, reduction of systemic and coronary plaque inflammation, and MACEs at 1 year of follow-up.

**Methods:**

In a multi-center study, we evaluated 369 T2DM patients with Mv-NOCS divided in 258 (69.9%) patients that did not receive the SGLT2-I therapy (Non-SGLT2-I users), and 111 (30.1%) patients that were treated with SGLT2-I therapy (SGLT2-I users) after percutaneous coronary intervention (PCI) and optical coherence tomography (OCT) evaluation. As the primary study endpoint, we evaluated the effects of SGLT2-I on FCT changes at 1 year of follow-up. As secondary endpoints, we evaluated at baseline and at 12 months follow-up the inflammatory systemic and plaque burden and rate of MACEs, and predictors of MACE through multivariable analysis.

**Results:**

At 6 and 12 months of follow-up, SGLT2-I users vs. Non-SGLT2-I users showed lower body mass index (BMI), glycemia, glycated hemoglobin, B-type natriuretic peptide, and inflammatory cells/molecules values (p < 0.05). SGLT2-I users vs. Non-SGLT2-I users, as evaluated by OCT, evidenced the highest values of minimum FCT, and lowest values of lipid arc degree and macrophage grade (p < 0.05). At the follow-up end, SGLT2-I users vs. Non-SGLT2-I users had a lower rate of MACEs [n 12 (10.8%) vs. n 57 (22.1%); p < 0.05]. Finally, Hb1Ac values (1.930, [CI 95%: 1.149–2.176]), macrophage grade (1.188, [CI 95%: 1.073–1.315]), and SGLT2-I therapy (0.342, [CI 95%: 0.180–0.651]) were independent predictors of MACEs at 1 year of follow-up.

**Conclusions:**

SGLT2-I therapy may reduce about 65% the risk to have MACEs at 1 year of follow-up, via ameliorative effects on glucose homeostasis, and by the reduction of systemic inflammatory burden, and local effects on the atherosclerotic plaque inflammation, lipids’ deposit, and FCT in Mv-NOCS patients with T2DM.

## Background

The sodium–glucose cotransporter 2 inhibitors (SGLT2-I) are hypoglycemic drugs, used in the treatment of patients with type 2 diabetes mellitus (T2DM), and exerting cardiovascular protective effects [1]. Indeed, the SGLT2-I could reduce the atherosclerotic plaque progression, instability, and rupture, via the significant downregulation of inflammatory burden [2, 3]. To date, SGLT2-I could significantly reduce major adverse cardiovascular events (MACEs) in T2DM patients with stable ischemic heart disease (IHD), as those with myocardial infarction [2, 3]. Conversely, the T2DM patients could have a relevant prevalence of multi-vessel non-obstructive coronary artery lesions (Mv-NOCS), which are prone to rupture [4]. Notably, the Mv-NOCS diabetic patients (particularly those with worse glycemic control), could report over-inflammation, lipids’ plaque accumulation and a higher burden of atherosclerotic plaque rupture with consequent MACEs [5]. Therefore, these factors are the main drivers of atherosclerotic plaque instability and lead to the reduction of fibrous cap thickness (FCT) [5]. From the current shreds of evidence, the FCT is recognized as valid marker of coronary stable vs. unstable plaque phenotype [5]. On the other hand, there is no conclusive data about the effects of SGLT2-I on atherosclerotic plaque and clinical outcomes in Mv-NOCS patients with T2DM. Conversely, less is reported about the effects of SGLT2-I therapy on inflammatory processes, lipids’ metabolism of atherosclerotic plaque, and modulation of FCT in Mv-NOCS patients with T2DM. In this setting, recently, authors found that SGLT2 protein is expressed at the level of peri-coronary fat and atherosclerotic plaque [6, 7]. Notably, SGLT2-I are implied in the modulation of inflammatory processes and lipids’ metabolism of atherosclerotic plaque [6, 7]. Indeed, the SGLT2-I (block of SGLT2-mediated pathways) could reduce the inflammatory burden at the coronary level and ameliorate the lipid profile [6, 7], thus reducing atherosclerosis process progression, leading to the best clinical outcomes [8].

In this setting, authors found that the SGLT2-I reduced the lipid accumulation in the adipose tissue and the liver production of atherogenic lipoproteins [9]. To date, the SGLT2-I could have a remarkable effect on lipid metabolism, and act by decreasing lipid accumulation, visceral and subcutaneous fat, and changing the body composition [10]. Conversely, the SGLT2-I also regulate key molecules in lipid synthesis and transportation and affect the oxidation of fatty acids [10]. Notably, atherogenic lipoproteins are a marker of increased atherosclerotic extension in prediabetes and diabetes, involved in diabetic dyslipidemia, and may be useful to identify subjects with a higher cardiovascular risk profile [11].

Therefore, we might speculate that the SGLT2-I could exert ameliorative effects on Mv-NOCS patients with T2DM and stable IHD, via significantly reducing inflammation and oxidate lipids’ coronary plaque accumulation and then leading to the increase of FCT values. These effects could lead to coronary plaque stability and to the significant reduction of MACEs in Mv-NOCS patients with T2DM. Thus, to investigate this study hypothesis, we evaluated, in a population of Mv-NOCS patients with T2DM, the effects of SGLT2-I therapy vs. other classes of oral anti-diabetic medications, in terms of changes in the plaque morphology, inflammatory burden, and MACEs at 12 months of follow-up.

## Methods

### Study patients and design

**Fig. 1:**
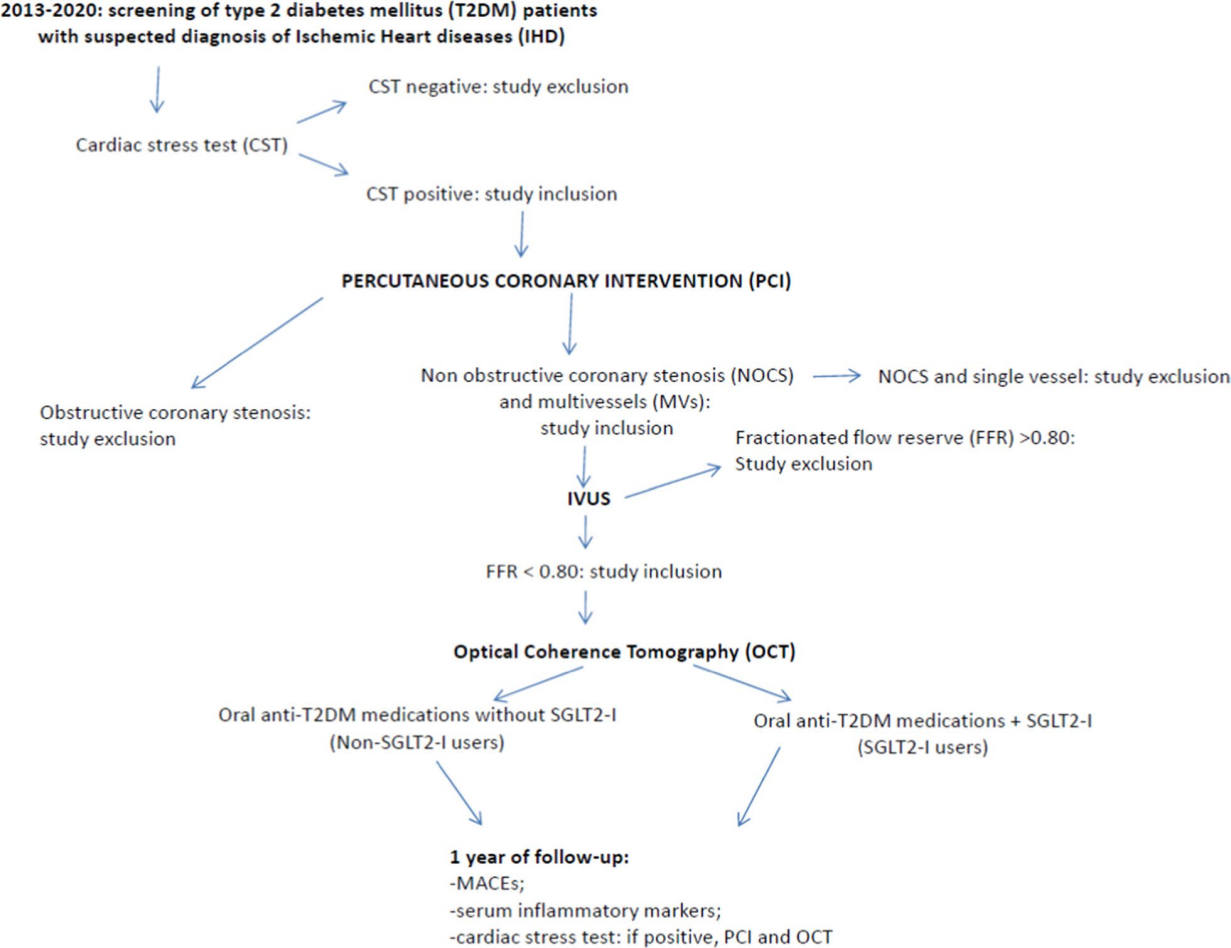
Study flow chart. *SGLT2-I* sodium glucose transporter 2 inhibitors, *MACEs* major adverse cardiac events

We conducted an observational, multicenter study, at the Department of Cardiology of the Cardarelli Hospital in Naples, at the Division of Cardiology” Città della Salute e della Scienza”, Turin, at the Department of Cardiology, “San Giovanni Bosco Hospital”, Turin, at the Unit of Cardiology, “S. Leonardo Hospital”, Castellammare, Naples, at Department of Cardio-Thoracic and Respiratory Sciences, University of Campania “Luigi Vanvitelli”, Caserta, and at Department of Cardiovascular Diseases, “Gemelli Molise”, Campobasso, Italy. From January 2013 to June 2021, we prospectively screened a population of consecutive 11,623 patients with evidence of stable ischemic heart disease (IHD), and positive cardiac stress test, admitted to hospital for receiving an elective coronary artery angiography. The patients did not evidence changes in the frequency, duration, or intensity of clinical symptoms within 4 weeks and were referred for elective coronary artery angiography (Fig. 1). Thus, 1258 patients with stable IHD, evidence of Mv-NOCS (20–49% luminal stenosis), and with negative fractional flow reserve (FFR > 0.80), entered the study (Fig. 1). Of these 428 patients with Mv-NOCS and FFR > 0.80, and a confirmed diagnosis of T2DM, were enrolled in the study (Fig. 1). The patients’ data were prospectively entered into a central database, collected, and revised at the Department of Advanced Medical and Surgical Sciences of the University of Campania “Luigi Vanvitelli”, Naples, Italy. From the current study we excluded patients with presence of obstructive and Mv-obstructive stenosis, patients with Mv-NOCS and FFR < 0.80, left ventricular ejection fraction less than 35%, previous myocardial infarction, previous percutaneous coronary intervention (PCI) or/and coronary bypass grafting, Tako-tsubo cardiomyopathy, myocarditis, acute or chronic infection or inflammatory diseases, hematologic disorder, malignancies, end-stage liver or renal disease, and use of steroid therapy or chemotherapy. From the current study, we also excluded patients with the diagnosis of left main trunk lesions, bifurcation lesions requiring two stents, cardiogenic shock, recommended coronary artery bypass grafting, severe chronic kidney disease, FFR > 0.80, and unsuccessful PCI (Fig. 1).

T2DM was diagnosed according to American Diabetes Association criteria [12]. All the T2DM patients with Mv-NOCS included in the study answered a specific questionnaire about medicines used for diabetes treatment before the beginning of the study, the dates of the beginning and the end of treatment, the route of administration, and the duration of use. We used the information from the medicine inventory during the study and this specific questionnaire to classify the subjects. However, we had finally 258 (69.9%) T2DM patients that did not receive the SGLT2-I therapy (Non-SGLT2-I users), and a cohort of 111 (30.1%) T2DM patients that were treated with SGLT2-I therapy (SGLT2-I users) after the procedure, according to international guidelines for the care of patients with T2DM and IHD [13] (Fig. 1). The enrolled patients were not randomized to the treatment with SGLT2-I vs. other classes of hypoglycemic drugs. Thus, we collected the data of the Non-SGLT2-I users that received the SGLT2-I (after 2019) and those that were not discharged without SGLT2-I after PCI and OCT (after 2013), according to the standard of care of T2DM [10], and of T2DM patients with IHD [14]. However, to assess the effect of SGLT2-I on the plaque morphology, we measured the FCT in the patients SGLT2-I users vs. Non-SGLT2-I users. The FCT was evaluated by optical coherence tomography (OCT), an intra-coronary imaging technique used to study atherosclerotic plaque morphology [15]. The OCT was repeated at 1 year of follow-up in patients with Mv-NOCS (SGLT2-I users vs. Non-SGLT2-I users), taking maximally tolerated statin therapy. The inflammatory burden was evaluated in SGLT2-I users vs. Non-SGLT2-I users, at baseline and at the follow-up end, via serum evaluation of molecular and cellular inflammatory markers. Finally, we reported the rate of MACEs in SGLT2-I users vs. Non-SGLT2-I users patients with Mv-NOCS at 1 year of follow-up. The study was conducted in accordance with the Declaration of Helsinki. The Ethics Committees of all participating institutions approved the protocol (the Ethic Committee University of Campania “Luigi Vanvitelli” number: 1177).

### Study endpoints

As the primary study endpoint, we evaluated in the SGLT2-I users vs. Non-SGLT2-I users after index procedure the effect of SGLT2-I on changes in FCT. The FCT is a parameter to describe the plaque morphology, and its modifications are the index of changes in the plaque morphology. The FCT was evaluated at baseline by OCT [16]. The OCT was then repeated at 1 year of follow-up in patients with Mv-NOCS and normal fractionated flow reserve (FFR > 0.80), taking maximally tolerated statin therapy. The patients with FFR > 0.80 were defined as those with normal coronary vessel flow reserve [17].

As secondary endpoints, we evaluated at baseline and at the follow-up end the circulating molecular and cellular pathways (inflammatory status), and at follow-up end the rate of MACEs. The MACEs were defined as a composite endpoint indicating cardiovascular disease events, hospital admissions for heart failure, and ischemic cardiovascular events [18]. We included in the diagnosis of cardiovascular disease events the diagnosis of ischemic heart disease, peripheral arterial disease, stroke/transitory ischemic attack, or revascularization procedure [18], according to the International Classification Codes-10. For the inflammatory activity, we evaluated at the molecular level: serum levels of NLR family pyrin domain containing 3 (NLRP3) inflammasome, caspase-1, and interleukin 1 beta (IL1β), which are defined as macro-inflammatory complex (ref). At the cellular level we evaluated the serum levels of CD86+ and CD 206+ cells’ surface antigens, marking the macrophage 1 and 2 (M1, M2) respectively [6–8].

### Clinical evaluation of study cohorts

#### Non-invasive stress test

The non-invasive stress tests (NITs) were performed at each participating center by a standard protocol via exercise treadmill test, exercise or dobutamine stress echocardiography, single-photon emission computed tomography, or positron emission tomography, as indicated by international guidelines [19, 20]. The involved physicians selected the NITS according to patient characteristics, local expertise, and availability. The physicians, blinded to the study cohorts, then reviewed and interpreted the NITs data. However, the results of NITs were reported as a binary variable, with a positive or negative result. We defined positive the NITs evidencing moderate to a severe reversible defect on nuclear perfusion imaging (≥ 10% ischemic myocardium) or high-risk findings on exercise treadmill test without imaging (≤ − 11 Duke Treadmill Score) [18–20].

#### Patients monitoring and clinical visits

Clinical evaluation included physical examination, vital signs, and review of adverse events, and the MACEs were collected during patients’ interviews, visits, and hospital discharge schedules. We reported all the events with the potential to be adjudicated as one of the predefined study endpoints, regardless of the investigator’s opinion. To date, in identifying a suspected unreported event by a reviewer, we asked the reviewer to make a note back to the investigator.

In the study cohorts, we performed fasting blood (at least 12 h from the last meal) for glycemia, lipid profile (total cholesterol (TC), triglycerides, high-density lipoprotein cholesterol (HDL-C), and low-density lipoprotein cholesterol (LDL-C) and B-type natriuretic peptide (BNP) at every visit. Syncope recurrence and other clinical events were collected during patients’ interviews, visits, and hospital discharge schedules. We made the diagnosis of T2DM according to international recommendations: plasma glucose values as fasting plasma glucose level ≥ 7.0 mmol/L (126 mg/dL), plasma glucose ≥ 11.1 mmol/L (200 mg/dL) either while fasting or not fasting, glycated hemoglobin ≥ 48 mmol/mol, and clinical history of diabetes and by the current use of anti-diabetic medications [14]. Thus, we defined the SGLT2-I users as the patients receiving SGLT2-I therapy before starting the study. The rest of the T2DM patients (without SGLT2-I therapy) were defined as Non-SGLT2-Iusers. The SGLT2-I users received either 10 mg or 25 mg of empagliflozin once daily, canagliflozin 100 mg daily, and/or dapagliflozin 10 mg daily in the last month before starting the study (mean duration of SGLT2-I therapy 27 ± 4.8 days at study beginning). The enrolled patients were regularly followed by clinical visits 10 days, 6th, and 12th months after clinical discharge by the treating physician.

#### Coronary angiography, fractional flow reserve (FFR), OCT image protocol and analysis

At hospital admission, all the patients were assigned to undergo coronary angiography, with the visualization of the left main trunk (LMT), left anterior descending (LAD), left circumflex (LCX), and right coronary artery (RCA). The coronary angiography was performed by interventional cardiologists, followed by PCI with the evaluation of intra-coronary FFR, and OCTof Mv-INOCS, respectively. After this, the cardiologists blinded to patient categorization (SGLT2-I users vs. Non-SGLT2-I users), reviewed selecting cases with Mv-INOCS, as coronary vessels with no-altered fractional flow reserve (FFR > 0.80), and associated to 20–49% luminal stenosis [17, 18]. We investigated the coronary flow reserve (CFR) via intravascular ultrasound (IVUS) and using an intracoronary Doppler guide advanced within the coronary infusion catheter and positioned in the mid-left anterior descending coronary artery [17]. The physicians, full trained in coronary angiography, measured the FFR using a 0.014-inch miniaturized pressure monitoring guide wire system, Pressure Wire (Radi Medical Systems), to record the coronary pressure distal to the segment. Thus, FFR was calculated by dividing the mean distal coronary pressure by the mean proximal coronary pressure, measured by the guiding catheter, during maximal hyperemia [17]. To date, we induced the hyperemia with intracoronary adenosine, at the recommended intracoronary dosage of 15 to 30 µg for the right coronary artery and 20 to 40 µg for the left coronary system, leaving the exact dosage to the operators’ discretion. The FFR calculations were acquired and then reviewed in a core laboratory by investigators blinded to the OCT results. Thus, as previously reported [17], we had a cut-off value of 0.80 to indicate Mv-INOCS without altered coronary flow reserve (FFR > 0.80), and Mv-INOCS with altered coronary flow reserve (FFR < 0.80).

Therefore, we defined Mv-INOCA as the patients with anginal symptoms, positive NITs, and evidence of functionally nonobstructive and Mv coronary disease [21]. On the contrary, we defined as No-INOCA the patients with anginal symptoms, negative NITs and functionally nonobstructive CAD [21].

Finally, in the Mv-INOCS patients, we performed the OCT using the frequency-domain OCT system (C7-XRTM Intravascular Imaging System and Dragonfly TM OCT catheter; St. Jude Medical, St. Paul, MN, USA) with a motorized pull-back system at 20 mm/s and rotation speed of 100 frames/s, using a non-occlusive technique. We registered and revised the OCT images at baseline, side by side. Thus, we matched the target lesions based on their distances from landmarks, such as branches and calcifications. The independent, experienced OCT investigators, and blinded to the patient groups, measured fibrous-cap thickness using a dedicated offline review system (St. Jude Medical) at the laboratory. We adjusted the calibration before OCT analysis and determined the minimum lumen area in each target lesion by an automated measurement algorithm and additional manual corrections. We characterized the plaque tissue according to validated criteria [22]. Furthermore, we identified the fibrous cap and the lipid of the necrotic core.

**Fig. 2:**
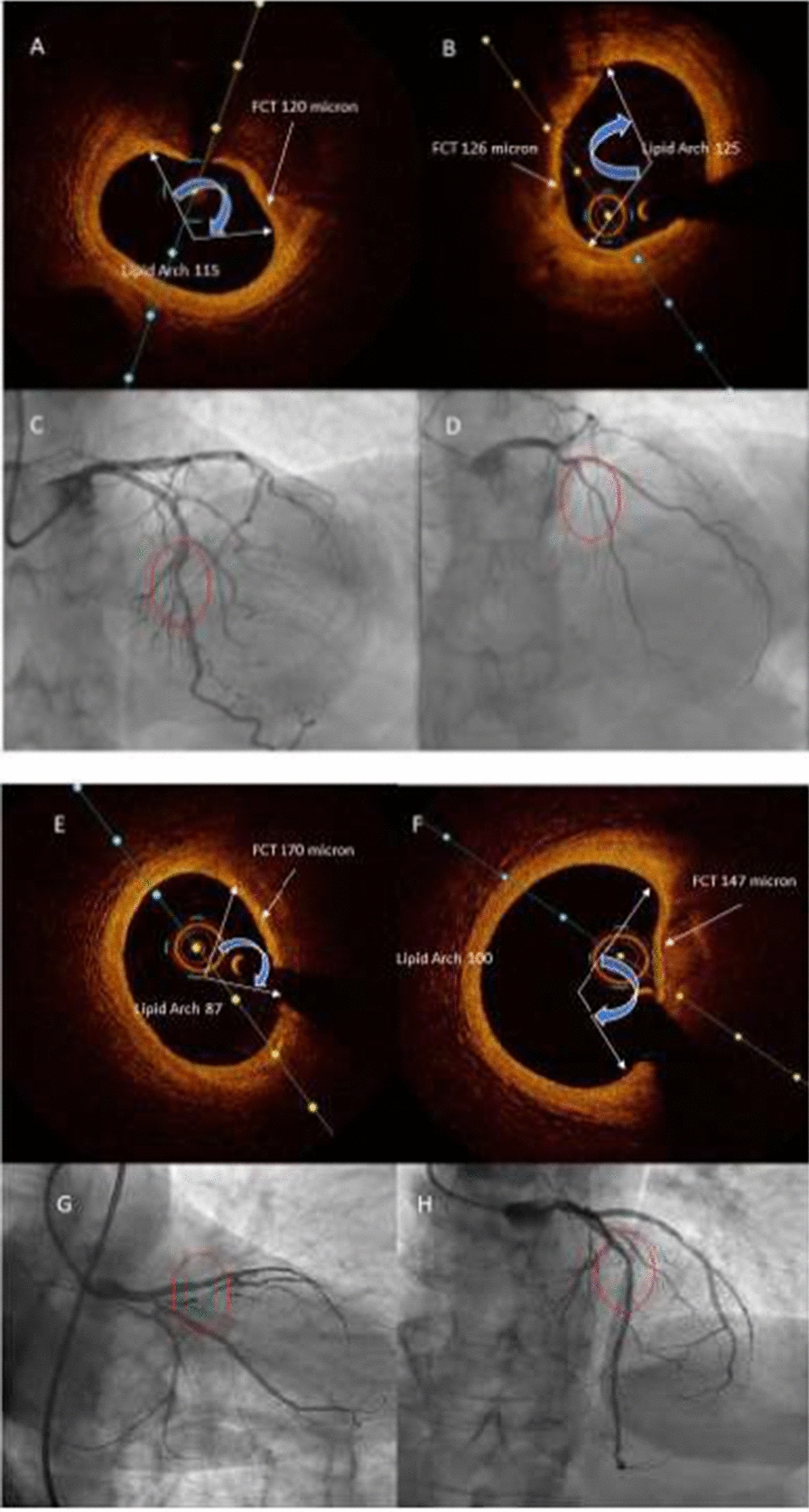
In the upper part of panel, representative images of the baseline values of fibrous cap thickness (FCT) and lipid arch in SGLT2-I users (**A**, left part) vs. Non-SGLT2-I users (**B**, right part) patients. The lipid length was 8.46 ± 1.87 vs. 8.39 ± 1.89 mm (p > 0.05) in SGLT2-I users vs. Non-SGLT2-I users patients. The macrophage grade was 12.04 ± 2.29 vs. 12.07 ± 2.31 (p > 0.05) in SGLT2-I users vs. Non-SGLT2-I users patients. The representative images of coronary vessels (inferior part) are of the middle segment of left anterior descending artery (LAD), comparing the SGLT2-I users (**C**, left part) vs. Non-SGLT2-I users’ (**D**, right part) patients. The red circle evidences the intermediate and non-obstructive coronary stenosis (NOCS) LAD stenosis. In the lower part of the panel, representative images of follow-up end values of fibrous cap thickness (FCT) and lipid arch in SGLT2-I users (**E**, left upper part) vs. Non-SGLT2-I users patients (**F**, right upper part). The lipid length was 7.92 ± 1.89 vs. 7.79 ± 1.88 mm (p > 0.05) in SGLT2-I users vs. Non-SGLT2-I users patients. The macrophage grade was 7.24 ± 2.22 vs. 9.27 ± 2.56 (p < 0.05) in SGLT2-I users vs. Non-SGLT2-I users patients. The representative images of coronary vessels (inferior left and right part) is the middle segment of anterior descending artery, comparing the SGLT2-I users (**G**, left inferior part) vs. Non-SGLT2-I users’ (**H**, right inferior part) patients. The red circle evidences the intermediate and non obstructive coronary stenosis (NOCS) LAD stenosis. *SGLT2-I* sodium glucose transporter 2 inhibitors

The fibrous cap was a lesion with high backscattering and relatively homogeneous OCT signal; the lipid or necrotic core, a signal-poor region with poorly delineated borders, little or no signal backscattering, and an overlying signal-rich layer, the fibrous cap. Finally, we calculated the minimum fibrous cap thickness [12, 16, 20–22]. We measured the fibrous cap thickness of each lipid-rich plaque, first at 1-mm intervals over the lipid plaque, then three times at its thinnest part at each cross-section, and then we calculated the average value [12, 16, 20–22]. We have determined the minimum fibrous cap thickness as the smallest fibrous cap thickness in the candidate frames (Fig. 2). We determined the maximum lipid arc as the largest lipid arc from the center of the lumen in the three candidate frames selected by visual screening (Fig. 2). We selected, for the measurements, a frame to be as similar as possible to the side branch and the lesion morphology, and the center of the lumen was determined to measure the largest lipid arc [12, 15, 20–22]. To date, we calculated the lipid length from the number of frames with lipid cores and performed macrophage semi-quantification on the same OCT cross-sections for qualitative plaque assessment, according to the OCT macrophage grading system, to semi-quantify the bright spots based on axial and circumferential distribution, as follows: grade 0, no macrophage; grade 1, localized macrophage accumulation; grade 2, clustered accumulation < 1 quadrant; grade 3, clustered accumulation 1 quadrant and < 3 quadrants; and grade 4, clustered accumulation 3 [12, 16, 20–22]. At follow-up end, we repeated the coronary angiography, with the evaluation of FFR, and the OCT in the Mv-NOCS patients. However, the OCT images were repeated to re-assess the atherosclerotic plaque morphology and the FCT of the atherosclerotic lesions implied in the clinical event in the SGLT2-I users vs. Non-SGLT2-I users, as previously indicated [12, 16, 20–22].

#### Echocardiographic assessment

Experienced physicians practiced the two-dimensional echocardiography in all hospital admitted patients, and in a blinded way to the groups of treatment. For the echocardiography the physicians used a standardized protocol and phased-array echocardiographs with M-mode, 2-dimensional, and pulsed, continuous-wave, and color flow Doppler capabilities [23]. The left ventricle ejection fraction was calculated from area measurements with the area-length method applied to the average apical area [23]. The left ventricular internal dimension and interventricular septal were measured at the end diastole and end systole, and the wall motion score index was calculated according to American Society of Echocardiography recommendations [23]. The physicians practiced this exam at hospital admission, and at 6 and 12 months of follow-up.

#### Serum collection and analysis of biochemical markers and inflammatory markers

The collection of patient blood samples was performed in an ice-cooled blood collection system at baseline (T0), after 24 h (T1), 6 months (T2) and 12 months (T3) from SGLT2-I users and Non-SGLT2-I users. We used a 21-gauge butterfly needle, inserted intravenously in the forearm, and after a 30-min supine rest, blood samples were drawn into chilled EDTA tubes, mixed with aprotinin, then divided into aliquots and stored at − 80 °C. Then, the samples were centrifuged for 10 min at 2.500 rpm at 4 °C and stored at − 80 °C. From the serum of study participants, we evaluated glycemia, glycated hemoglobin (Hb1Ac), other biochemical markers, high sensitivity T troponin (hs-TnT), and BNP, after completing an overnight fast except for taking their regularly prescribed medications. To measure the BNP serum values, we used the Triage B-type natriuretic fluorescence immunoassay (Biosite Diagnostics) in frozen plasma samples thawed to room temperature. To date, we fixed the lowest detectable measurement for this assay at 5 pg/mL, WITH an inter-assay coefficient of variation of 10.1% for 28.8 pg/mL, 12.4% for 586 pg/mL, and 16.2% for 1180 pg/mL. To evaluate the inflammatory burden in SGLT2-I vs. Non-SGLT2-I users, we evaluated the serum levels of NLRP3 Inflammasome, Caspase-1, Interleukin-1β (IL-1β), CD86 as a marker of M1 macrophages and CD206 as a marker of M2 macrophages. We used the commercially available kits according to the manufacturer’s protocols (MBS3802246 MyBioSource; ELH-CASP1-1 RayBiotech; ab214025 Abcam; ab45921 Abcam; MBS2604362 MyBioSource). OD values were determined by using an ELISA plate reader.

### Follow-up

After discharge from the hospital, all patients were managed and followed at 6th and 12th month (follow-up end) after the event as outpatients to perform clinical evaluation, routine analyses, and cardiovascular evaluation, as well as, with the goal to maintain HbA1c level at < 7%, fasting blood glucose level of 90–140 mg/dL and post-prandial blood glucose level of < 180 mg/dL.

### Statistical analysis

A qualified statistician revised and analyzed the collected data and verified the normality (Shapiro–Wilk’s test) and equal variance (Bartlett’s test) of study variables. Thus, the continuous variables, expressed as means and standard deviations, were tested by a two-tailed Student T-test or Mann–Whitney. The categorical variables were compared by Chi-square or Fisher exact test, as appropriate. We performed the survival analysis using the Kaplan–Meier method. We evaluated the predictors of MACEs by using Cox regression models adjusted for potential confounders. We conducted a univariate analysis to examine the association between hypertension, smoking, HbA1c, interleukin-6 (IL-6), minimum FCT, macrophage grade, SGLT2-I therapy, and MACEs at 12 months of follow-up. All variables with a p-value of less than 0.2 in the univariate analysis were subsequently entered into a multivariate model. In the multivariate model, a p-value of less than 0.05 was considered statistically significant. For all independent predictors, 95% confidence intervals were calculated. Statistical significance was established at a p-value < 0.05 for all the other analyses. The statistical analysis was performed using the SPSS software package (SPSS Inc., Armonk, NY).

## Results

In the current study, we evaluated at 1 year of follow-up the effects of SGLT2-I therapy vs. other class of oral anti-diabetic medications in the study cohorts (SGLT2-I users vs. Non-SGLT2-I users) at clinical, cellular and molecular level, and in terms of primary and secondary study endpoints. At baseline, the SLGT2-I users (n 111) vs. Non-SGLT2-I users (n 258) did not show significant difference regards the clinical characteristics, the inflammatory markers, the drugs’ therapy, and OCT data (Table 1). At baseline, by comparing the SLGT2-I users vs. Non-SGLT2-I users, we did not evidence significant differences regards the parameters evaluated by coronary angiography, as the target lesion [LAD: 42 (37.8%) vs. 95 (36.8%); LCX: 19 (17.1%) vs. 52 (20.2%); RCA: 50 (45%) vs. 111 (43%); p 0.733], lumen area (11.96 ± 2.80 vs. 11.58 ± 2.58 mm2; p 0.123), mean reference diameter (2.65 ± 0.55 vs. 2.59 ± 0.48 mm; p 0.325), percentage stenosis diameter (45.12 ± 11.25 vs. 43.56 ± 10.94%; p 0.215), and FFR (0.82 ± 0.65 vs. 0.83 ± 0.58%; p 0.364). At 6 months of follow-up, the SGLT2-I users vs. Non-SGLT2-I users, showed lower values of body mass index (BMI), glycemia, Hb1Ac, BNP, and inflammatory cells (white blood cells, granulocytes, monocytes), and molecules [C reactive protein (CRP), IL-6, tumor necrosis factor alpha (TNFα), nitrotyrosine] (p < 0.05; Table 1).

**Table 1:** Clinical characteristics, inflammatory data, optical coherence tomography (OCT) data and drug therapy at baseline and follow-up in the study cohorts

Clinical characteristics	Baseline			Six months			Twelve months		
	SGLT2-I users (n 111)	Non-SGLT2-I users (n 258)	P value	SGLT2-I users (n 111)	Non-SGLT2-I users (n 258)	P value	SGLT2-I users (n 111)	Non-SGLT2-I users (n 258)	P value
Age, years	66.4 ± 5.5	65.4 ± 5.9	0.342	–	–		–	–	
Male, n (%)	62 (56.9)	149 (57.8)	0.819	–	–		–	–	
BMI (kg/m^2^)	28.2 ± 1.6	28.5 ± 1.9	0.126	27.5 ± 1.2	28.2 ± 1.8	0.001*	27.3 ± 0.9	28.1 ± 1.3	0.001*
Current smokers, n (%)	13 (11.7)	33 (12.8)	0.864	11 (9.9)	31 (12.0)	0.559	10 (9.0)	27 (10.5)	0.669
Dyslipidemia (%)	58 (52.3)	147 (57)	0.425	62 (55.9)	157 (60.9)	0.370	64 (57.7)	163 (63.2)	0.318
Hypertension, n (%)	69 (62.2)	168 (65.1)	0.636	73 (65.5)	177 (68.6)	0.593	77 (69.4)	184 (71.3)	0.706
Glycaemia (mg/dL)	198.8 ± 24.6	194.1 ± 26.5	0.108	148.5 ± 35.2	162.4 ± 27.4	0.001*	138.1 ± 21.4	150.1 ± 27.3	0.010*
Hb1Ac (%)	6.5 ± 0.3	6.4 ± 0.2	0.169	5.8 ± 0.4	6.1 ± 0.5	0.011*	5.5 ± 0.3	6.0 ± 0.4	0.001*
Cholesterol (mg/dL)	203.5 ± 21.5	207.2 ± 19.1	0.108	188.2 ± 23.2	190.1 ± 19.1	0.496	183.1 ± 19.6	184.4 ± 13.9	0.516
HDL	38.2 ± 3.2	37.9 ± 3.4	0.327	44.4 ± 3.7	43.8 ± 3.9	0.268	48.1 ± 5.7	47.5 ± 6.1	0.221
LDL	128.7 ± 21.9	129.5 ± 18.7	0.324	114.6 ± 19.6	117.9 ± 17.3	0.107	105.3 ± 16.3	108.0 ± 14.4	0.125
Heart rate (bpm)	84.8 ± 7.5	85.4 ± 9.1	0.559	80.2 ± 8.7	80.7 ± 10.0	0.582	71.9 ± 8.7	72.3 ± 9.8	0.602
Systolic BP (mmHg)	127.4 ± 7.9	126.1 ± 10.6	0.226	124.8 ± 8.5	125.3 ± 10.2	0.651	123.5 ± 8.3	124.8 ± 10.3	0.235
Diastolic BP (mmHg)	79.7 ± 7.5	78.9 ± 7.3	0.349	77.8 ± 8.6	78.9 ± 7.3	0.222	77.3 ± 8.1	78.4 ± 6.5	0.170
Creatinine (mg/dL)	0.97 ± 0.15	0.98 ± 0.20	0.549	1.02 ± 0.15	1.04 ± 0.16	0.919	1.10 ± 0.13	1.09 ± 0.16	0.816
hs-TnT (ng/mL)	1.01 ± 0.51	1.09 ± 0.70	0.193	0.87 ± 0.28	0.95 ± 0.33	0.160	0.66 ± 0.19	0.69 ± 0.24	0.455
BNP (pg/mL)	45.9 ± 30.4	48.3 ± 34.4	0.536	36.5 ± 27.4	51.7 ± 33.6	0.001*	31.1 ± 20.6	55.4 ± 25.2	0.001*
LVEF (%)	58.2 ± 3.9	58.0 ± 4.2	0.488	58.0 ± 4.0	57.8 ± 4.2	0.744	58.6 ± 4.8	58.2 ± 6.0	0.127
Inflammatory markers									
WBC (×10^6^)	7.63 ± 0.58	7.72 ± 0.76	0.293	7.48 ± 0.65	7.84 ± 0.68	0.001*	7.27 ± 0.62	7.87 ± 0.69	0.001*
Granulocytes (×10^6^)	4.62 ± 0.70	4.74 ± 0.62	0.121	4.45 ± 0.70	4.85 ± 0.61	0.001*	4.27 ± 0.62	5.02 ± 0.33	0.001*
Monocytes (×10^3^)	0.37 ± 0.03	0.38 ± 0.05	0.079	0.39 ± 0.04	0.41 ± 0.06	0.012*	0.39 ± 0.04	0.42 ± 0.06	0.001*
Platelets (×10^3^)	267.23 ± 28.11	269.71 ± 29.13	0.449	247.14 ± 31.7	252.43 ± 32.26	0.213	234.71 ± 37.18	238.21 ± 40.88	0.439
Fibrinogen (mg/dL)	327.90 ± 32.34	333.59 ± 36.64	0.141	320.93 ± 32.36	326.12 ± 64.67	0.132	318.93 ± 33.42	324.30 ± 34.74	0.144
CRP (mg/dL)	2.45 ± 1.28	2.36 ± 0.78	0.389	2.28 ± 1.11	3.46 ± 0.62	0.001*	2.01 ± 0.80	3.69 ± 0.72	0.001*
IL-6 (pg/dL)	365.82 ± 45.51	352.49 ± 87.50	0.130	269.29 ± 45.51	282.72 ± 63.69	0.041*	250.33 ± 69.09	271.65 ± 66.01	0.002*
TNFα (mg/dL)	3.59 ± 1.12	3.51 ± 1.01	0.367	3.42 ± 0.92	5.33 ± 0.80	0.001*	3.36 ± 1.3	5.55 ± 0.76	0.001*
Nitrotyrosine (pg/dL)	0.26 ± 0.08	0.25 ± 0.06	0.258	0.37 ± 0.12	0.50 ± 0.10	0.001*	0.43 ± 0.12	0.54 ± 0.11	0.001*
OCT data									
Minimum FCT (µm)	126.09 ± 23.88	128.39 ± 18.98	0.326	–	–		170.29 ± 23.88	163.19 ± 18.98	0.003*
Lipid arc (°)	134.55 ± 30.49	132.82 ± 23.17	0.550	–	–		96.17 ± 30.49	109.82 ± 23.17	0.002*
Lipid length (mm)	8.68 ± 1.98	8.40 ± 1.90	0.202	–	–		8.08 ± 1.99	7.79 ± 1.90	0.202
Macrophage grade	12.04 ± 2.35	12.18 ± 2.43	0.609	–	–		6.97 ± 2.35	9.60 ± 2.88	0.001*
Drug therapy									
BBs, n (%)	42 (37.8)	102 (39.5)	0.759	45 (40.5)	110 (42.6)	0.976	46 (41.4)	112 (43.4)	0.726
CCBs, n (%)	26 (23.4)	65 (25.2)	0.717	29 (26.1)	74 (28.7)	0.616	32 (28.8)	78 (30.2)	0.787
ACEI, n (%)	28 (25.5)	64 (34.8)	0.932	29 (26.1)	66 (25.6)	0.913	29 (26.1)	70 (27.1)	0.842
ARB, n (%)	26 (23.4)	62 (24.0)	0.961	27 (24.3)	66 (25.6)	0.799	28 (25.2)	71 (27.5)	0.648
Statins, n (%)	58 (52.3)	147 (57)	0.425	69 (62.2)	175 (67.8)	0.291	73 (65.8)	180 (69.8)	0.448
ASA (%)	67 (60.4)	151 (58.5)	0.743	89 (80.2)	203 (78.8)	0.745	94 (84.7)	212 (82.2)	0.556
Clopidrogel (%)	21 (18.9)	46 (17.8)	0.803	24 (21.6)	54 (20.9)	0.881	26 (23.4)	59 (22.9)	0.908
DAPT (%)	17 (15.3)	36 (14.0)	0.732	93 (83.8)	218 (84.5)	0.863	94 (84.7)	223 (86.4)	0.658
Loop diuretics, n (%)	13 (11.7)	35 (13.6)	0.627	15 (13.5)	38 (14.7)	0.760	16 (14.4)	38 (14.7)	0.937
Tyazides, n (%)	11 (9.9)	27 (10.5)	0.872	13 (11.7)	31 (12.0)	0.934	15 (13.5)	33 (12.8)	0.867
Insulin, n (%)	13 (11.7)	28 (10.9)	0.810	14 (12.6)	31 (12.0)	0.876	15 (13.5)	37 (14.3)	0.834
Metformin, n (%)	48 (43.2)	110 (42.6)	0.914	50 (45.0)	119 (46.1)	0.849	52 (46.8)	124 (48.1)	0.829
Incretins, n (%)	32 (29.1)	79 (30.6)	0.78	36 (29.8)	85 (32.9)	0.923	41 (36.9)	97 (37.6)	0.415
DPP4-I	23 (71.8)	52 (65.8)	7	25 (69.4)	61 (71.8)		29 (70.7)	70 (72.2)	
GLP-1 RA	9 (28.2)	27 (34.2)		11 (30.6)	24 (28.2)		12 (29.3)	27 (27.8)	

*BMI* body mass index, *Hb1Ac* glycated hemoglobin, *HDL* high density lipoprotein, *LDL* low density lipoprotein, *BP* blood pressure, *hs-TnT* high sensitivity troponin T, *BNP* B type natriuretic peptide, *LVEF* left ventricle ejection fraction, *WBC* white blood cells, *CRP* C reactive protein, *IL-6* interleukin 6, *TNFα* tumor necrosis factor alpha, *OCT* optical coherence tomography, *FCT* fibrous cap thickness, *BBs* beta blockers, *CCBs* calcium blockers, *ACEI* angiotensin converting enzyme inhibitors, *ARB* angiotensin receptor blockers, *ASA* acetylsalicylic acid, *DAPT* dual anti-platelet therapy, *DPP4-I* dipeptidyl peptidase 4 inhibitors, *GLP-1 RA* glucagon-like peptide-1 receptor agonists, *SGLT2-I* sodium glucose transporter 2 inhibitors

*Is for statistical significant (p < 0.05) vs. Non-SGLT2-I users

At 12 months of follow-up, the SGLT2-I users vs. Non-SGLT2-I users showed lowest values of BMI, glycemia, Hb1Ac, and BNP (p < 0.05; Table 1). The SGLT2-I users vs. Non-SGLT2-I users had lowest values of inflammatory cells (white blood cells, granulocytes, monocytes), and molecules (CRP, IL-6, TNFα, nitrotyrosine), (p < 0.05; Table 1). The SGLT2-I users vs. Non-SGLT2-I users, as evaluated by OCT, evidenced highest values of minimum FCT, and lowest values of lipid arc degree and macrophage grade (p < 0.05; Table 1). Notably, at follow-up end, we noted a lower rate of MACEs comparing the SGLT2-I users vs. Non-SGLT2-I users [n 12 (10.8%) vs. n 57 (22.1%); p < 0.05].

**Fig. 3:**
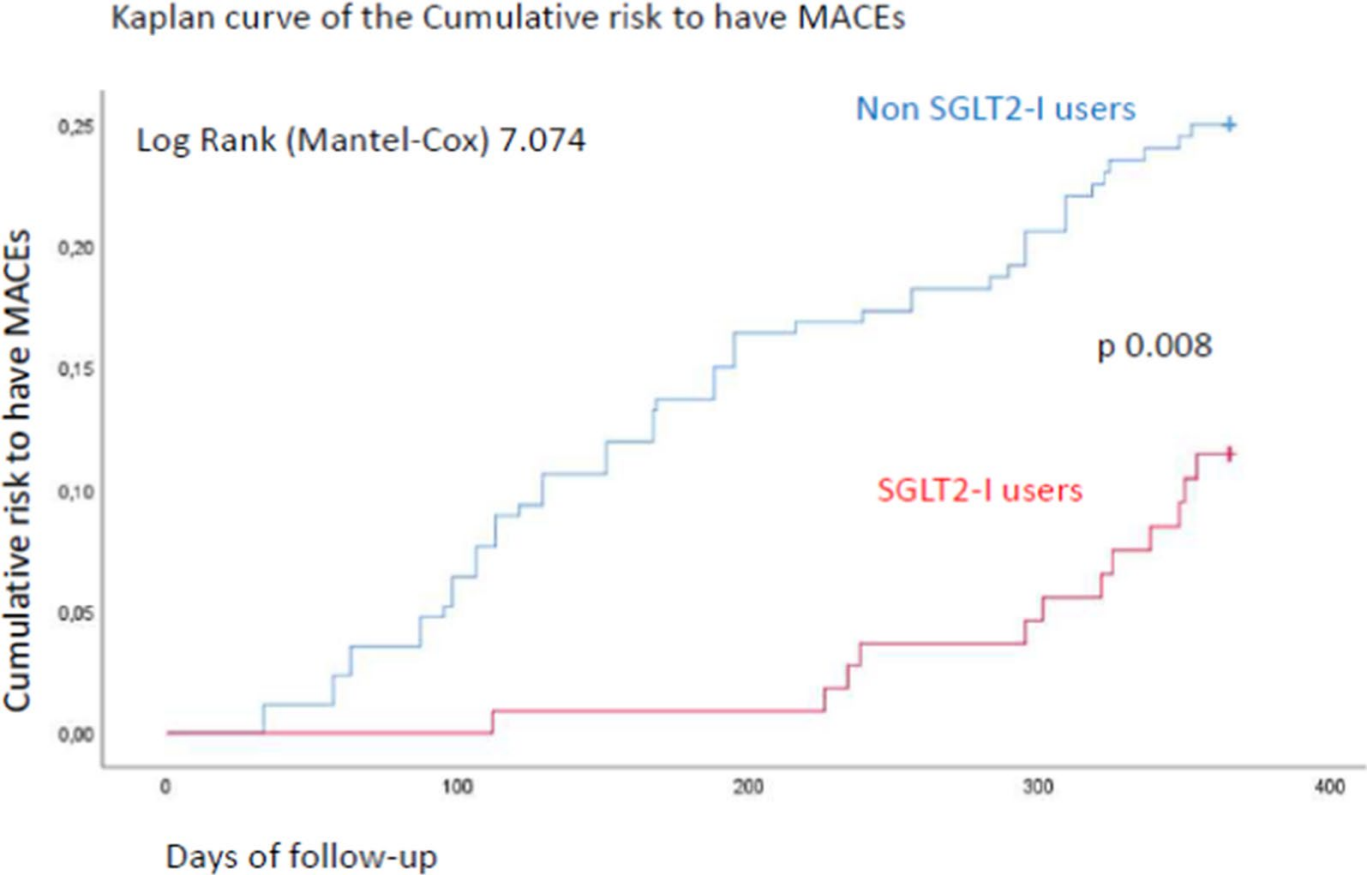
Kaplan curves of the cumulative risk to have MACEs at 1 year of follow-up in SGLT2-I users vs. Non-SGLT2-I users. *MACEs* major adverse cardiac events,* SGLT2-I* sodium glucose transporter 2 inhibitors

Finally, the Cox regression analysis showed that Hb1Ac values (1.930, [CI 95%: 1.149–2.176]), macrophage grade (1.188, [CI 95%: 1.073–1.315]), and SGLT2-I therapy (0.342, [CI 95%: 0.180–0.651]) were independent predictors of MACEs at 1 year of follow-up (Table 2). The Kaplan curve shows the cumulative risk of having MACEs at 1 year of follow-up in SGLT2-I users vs. Non-SGLT2-I users (Fig. 3).

**Table 2:** Multivariable Cox regression analysis for assessing the independent predictors of MACEs at 1 year of follow-up

Risk factors	Univariate analysis			Multivariate analysis		
	HR	95% CI	p value	HR	95% CI	p value
Hypertension	1.566	0.976–2.514	0.063			
Smoking	2.489	0.907–6.831	0.077			
HbA1c	3.100	2.206–9.854	0.001*	1.930	1.149–2.176	0.001*
IL-6	0.998	0.994–1.010	0.205			
Minimum FCT	1.021	1.008–1.034	0.001*	1.007	0.994–1.020	0.306
Macrophage grade	1.244	1.140–1.357	0.001*	1.188	1.073–1.315	0.001*
SGLT2-I	0.271	1.218–4.232	0.001*	0.342	0.180–0.651	0.001*

The total number of MACEs was 69 [n 12 (10.8%) in the SGLT2-I users vs. n 57 (22.1%) in the Non-SGLT2-I users; p < 0.05]

*MACEs* major adverse cardiac events, *HbA1c* glycated hemoglobin, *IL-6* interleukin 6, *FCT* fibrous cap thickness, *SGLT2-I* sodium glucose transporter 2 inhibitors

*Statistical significant (p < 0.05)

**Fig. 4:**
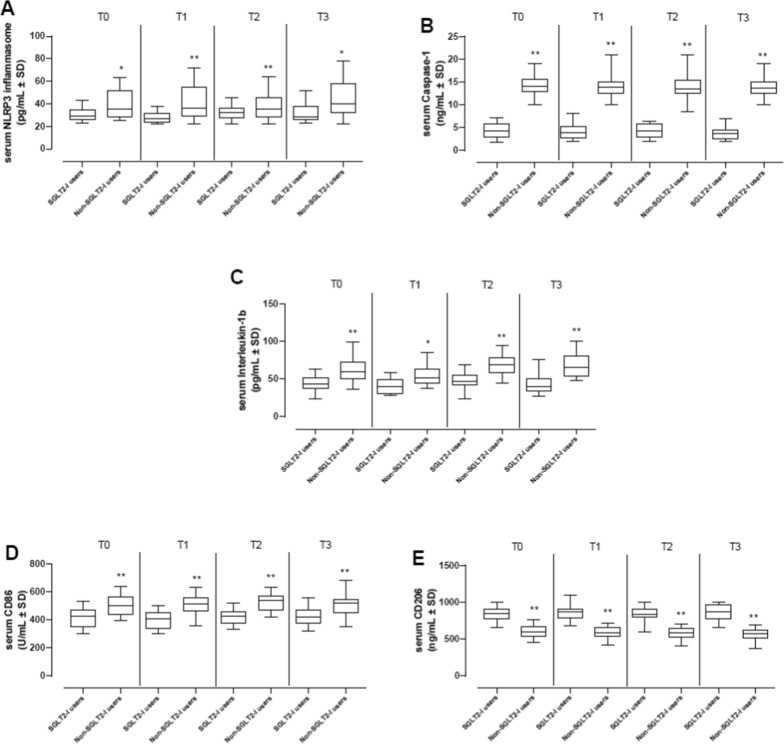
Plot boxes of serum inflammatory molecules and cells in the study cohorts. Serum levels of NLRP3 inflammasome (pg/mL), Caspase-1 (ng/mL) and Interleukin-1β (pg/mL) in diabetic SGLT2-I users (N = 111) and diabetic Non-SGLT2-I users (N = 258) at baseline (T0), after 24 h (T1), 6 months (T2) and 12 months (T3) of follow-up (**A**–**C**). Serum levels of CD86 as a marker of M1 macrophages (U/mL) and CD206 (ng/mL) in diabetic SGLT2-I users (N = 111) and diabetic Non-SGLT2-I users (N = 258) patients at baseline (T0), after 24 h (T1), 6 months (T2) and 12 months (T3) of follow-up (**D**, **E**). Values are reported as mean ± SD. *NLPR3* NLR family pyrin domain containing 3 (NLRP3) inflammasome, *SGLT2-I* sodium glucose transporter 2 inhibitors. **P < 0.01 vs SGLT2 users at same T; ^^P < 0.01 vs SGLt2 non-users at baseline

About the evaluation of serum molecular and cellular inflammatory burden, we found that at the T0 (baseline), the serum levels of NLRP3 inflammasome (31 ± 6 vs. 34 ± 8 pg/mL, p > 0.05), caspase-1 (4 ± 1 vs. 4 ± 2 ng/mL, p > 0.05), and IL-1β (44 ± 10 vs. 42 ± 10 pg/mL, p > 0.05) were not different in SGLT2-I users compared to Non-SGLT2-I users (Fig. 4).

At the T1, the SGLT2 non-users exhibited a significant increment of all the serum markers compared to SGLT2 users at the same time point, but also when compared to SGLT2 non-users at the T0 (p < 0.05; Fig. 4). The same trend was evident also at T2 and T3 (6 and 12 months of follow-up) (p < 0.05). Particularly, serum NLRP3 levels were higher in SGLT2 non-users at T1 (42 ± 15 pg/mL, p < 0.01 vs. SGLT2 users), T2 (36 ± 11 pg/mL, p < 0.01 vs. SGLT2 users) and T3 (44 ± 16 pg/mL, p < 0.05 vs. SGLT2 users; Fig. 4). Similarly, serum caspase-1 levels were increased in SGLT2 non-users compared to SGLT2 users at T1 (14 ± 2.7 ng/mL, p < 0.01), T2 (14 ± 2.5 ng/mL, p < 0.01) and T3 (14 ± 2 ng/mL, p < 0.01), as well as serum IL-1β levels (T1:55 ± 13 pg/mL, p < 0.05; T2:68 ± 13 pg/mL, p < 0.01; T3:69 ± 16 pg/mL, p < 0.01; Fig. 4).

About the serum markers of M1/M2 macrophage polarization (expression of inflammatory cells), comparing the SGLT2-I users to Non-SGLT2-I users at T0, we did not find significant difference about serum levels of CD86, as a marker of M1 macrophages (415 ± 68 vs. 412 ± 72 U/mL, p > 0.05), and serum levels of CD206, as a marker of M2 macrophages (835 ± 97 vs. 785 ± 100 ng/mL, p > 0.05; Fig. 4). Starting from T1, the Non-SGLT2-I users exhibited higher serum levels of CD86 compared to SGLT2 users (p < 0.05; Fig. 4). These were paralleled by lower levels of CD206 in Non-SGLT2-I users at all the subsequent time points (p < 0.05; Fig. 4). Particularly, Non-SGLT2 users at T1 exhibited serum CD86 levels of 511 ± 71 U/mL and CD206 levels of 591 ± 75 ng/mL (both p < 0.01 vs. SGLT2 users), which were similar at T2 (CD86: 525 ± 64 U/mL and CD206: 583 ± 83 ng/mL, both p < 0.01 vs. SGLT2 users) at T3 (CD86: 510 ± 74 U/mL and CD206: 559 ± 81 ng/mL, both p < 0.01 vs. SGLT2 users; Fig. 4).

## Discussion

In the current study, SGLT2-I users vs. Non-SGLT2-I users showed, from the 6 months of treatment, a significant reduction of BMI, and the amelioration of glucose homeostasis, BNP values, and inflammatory burden (cells/cytokines). At the follow-up end, we confirmed this ameliorative trend in SGLT2-I users vs. Non-SGLT2-I users. Notably, the SGLT2-I users vs. Non-SGLT2-I users showed a more significant increase of minimum FCT, and reduction of lipid arc degree and macrophage grade at follow-up, just receiving the same tolerated dose of maximal anti-lipids therapy. This was linked to a lower rate of MACEs, which were unfavorably increased in patients with worse glycemic control (Hb1Ac values, HR 1.930), higher macrophage grade (HR 1.188), and significantly reduced by the SGLT2-I therapy (HR 0.342) at 1 year of follow-up. Conversely, we found that at 6 and 12 months of follow-up (T1 and T2), the SGLT2-I users vs. Non-SGLT2-I users had the lowest expression of NLRP3 levels, serum caspase-1 and IL-1β levels (inflammatory markers) and stage of M1/M2 macrophage polarization, as serum levels of CD86 and CD206. In the current study, the SGLT2-I users’ patients differed regards those receiving 10 mg vs. 25 mg daily of empagliflozin, a third group receiving canaglifozin 100 mg daily, and a fourth group of patients under dapaglifozin 10 mg daily. On the other hand, the two-dose groups for empaglifozin, and the group under canaglifozin and dapaglifozin had a similar hazard ratio for cardiovascular outcomes. Thus, we might confirm the cardio-protective effects of the SGLT2-I, in the T2DM with MACEs recurrence.

Therefore, SGLT2-I therapy could exert anti-inflammatory effects and lead to a more stable atherosclerotic coronary plaque phenotype, as reported by the increase of minimum FCT and the reduction of plaque inflammation (macrophage grade) and lipids’ deposit (lipid arc degree) at follow-up end. Previous studies reported the systemic anti-inflammatory effects of SGLT2-I in cohorts of Mv-T2DM, as in those with coronary atherosclerotic plaque rupture and acute myocardial infarction [2, 3]. These effects caused a significant reduction of MACEs in SGLT2-I users vs. Non-SGLT2-I users [2, 3]. Authors previously found that the worse glycemic homeostasis could cause over-inflammation and worse prognosis via the increased expression of SGLT2 receptors and atherosclerotic plaque instability in the Mv-patients [2, 6]. As seen in humans’ ex vivo models, highest levels of inflammatory cytokines linked to the over-expression of the SGLT2 receptors al level of peri-coronary fat [6], and atherosclerotic plaque [7]. Conversely, the hypoglycemic drugs could down-regulate the SGLT2 pathways, reducing the over-inflammation (higher serum values of IL-1, IL-6, and TNF-α) in the SGLT2-I users vs. non-SGLT2-I users’ patients, and leading to best clinical outcomes [6]. In rats’ models, the block of the SGLT2 pathways by empagliflozin (SGLT2-I) reduced the inflammatory/oxidative stress in the non-infarcted myocardium and the mortality, acting by the protective modification of cardiac energy metabolism [24]. In humans, the canaglifozin (SGLT2-I) caused either a glucose-independent up-regulation of cardiac survival pathways leading to cardioprotective effects in high-risk cardiovascular patients irrespective of diabetic status [25], and reduced the inflammatory burden and myocardial size in patients with atherosclerotic plaque rupture and acute myocardial infarction [3]. Indeed, the SGLT2-I could ameliorate glucose homeostasis, lowering blood pressure, weight loss, and improving vascular and coronary function [3]. Intriguingly, SGLT2-I could exert cardiac protection beyond glucose and lipid-metabolic regulation [26]. Therefore, SGLT2-I cardioprotective properties could result from both a direct effect on glucose level reduction (glucose-lowering dependent effects) and a glycemic-independent effect, via the inhibition of the NLRP3 inflammasome [3]. Indeed, the SGLT2-I increased the plasma beta-hydroxybutyrate with a parallel decline in fasting plasma insulin levels due to a considerable improvement in insulin sensitivity via the inhibition of NLRP3 inflammasome activity [3]. Conversely, SGLT2-I might regulate coronary endothelial function via the modulation of autonomic tone in humans, platelet aggregation, lipoprotein, and glycemic metabolism [3, 27]. In this setting, the stage of lipids’ contents and inflammation, as assessed here by OCT, could characterize the coronary plaques prone to rupture [28]. Indeed, atherosclerotic plaques are characterized by larger lipid burden and lipid content, and thin fibrous caps are those unstable, with a higher rate of rupture and consequent clinical events [28]. In this context, authors found that the addition of the lipids’ lowering agents (PCSK9 inhibitor) to high-intensity statin therapy in patients with acute myocardial infarction, reducing the blood lipids values significantly, resulted in favorable effects on coronary atherosclerosis [28]. These ameliorative effects on the atherosclerotic plaque included a greater reduction in lipid burden and greater increase in minimal FCT, as assessed by OCT [28]. Similarly, among patients with stable IHD, those treated with evolocumab (PCSK9 inhibitor) vs. placebo achieved lower LDL-C levels, with a greater decrease in percent atheroma volume [29]. Thus, among patients with the angiographic coronary disease treated with statins, the addition of evolocumab, compared with placebo, reduced coronary plaque progression by lowering effects on LDL and coronary atheroma volume [29]. On the other hand, atherosclerotic plaques with large lipid pools, thin fibrous caps, and marked inflammatory cell infiltration are prone to rupture, potentially triggering fatal coronary events [30]. However, according to these observations, authors proposed the FCT as a marker of plaque stabilization [5, 28–30]. In our study, the worse glycemic control (highest Hb1Ac values), and the atherosclerotic coronary plaque over-inflammation (higher macrophage grade) could increase about 1.9-folds and 1.2-folds respectively the risk of having MACEs in Mv-INOCS patients with T2DM. From a current study, in the T2DM patients with FFR-negative coronary lesion (i.e. FFR > 0.80) that underwent OCT assessment, the TCF lesions are associated with a fivefold higher rate of MACE [31]. In fact, these lesions in diabetic cohorts could show a significantly higher prevalence of macrophage infiltration and a higher inflammation level that might eventually lead to fibrous cap destabilization and plaque rupture [31]. Furthermore, in T2DM patients the identification of TCF could be seen more important than ruling out the presence of flow-limiting lesions in predicting future cardiovascular events [31]. On the contrary, we might suggest that the therapy with SGLT2-I is an inverse predictor of about 65% of the risk of having MACEs in Mv-NOCS patients with T2DM. These study results could confirm the hypothesis of the worse glycemic control (highest Hb1Ac values) as the main cause of atherosclerotic plaque over-inflammation (highest macrophage grade), and rupture with consequent worse clinical outcomes (increased rate of MACEs). Thus, we might speculate that the SGLT2-I ameliorative clinical effects could be due to stabilizing properties on the atherosclerotic plaque. Indeed, SGLT2-I could reduce the lipids’ plaque metabolism (lipid arc degree) and inflammation (macrophage grade) and increase the FCT values. Indeed, from current studies, the increase in minimal FCT values could identify patients with a more stable coronary plaque phenotype [28–31]. Furthermore, our study provides evidence about the effects of SGLT2-I on systemic and local plaque inflammation, added to the reduction of lipids accumulation in the Mv-NOCS lesions and the increase of FCT values. Thus, the SLGT2-I might induce the stabilization of atherosclerotic plaque in Mv-NOCS diabetic patients, leading to the best clinical outcomes (reduction of MACEs). However, the SGLT2-I might exert pleiotropic effects on atherosclerotic cap inflammation, lipids accumulation, and FCT in Mv-NOCS patients. Therefore, we might propose SGLT2-I as drugs with stabilizing effects on the atherosclerotic plaque via anti-inflammatory properties, reduced lipids’ accumulation, and increased FCT. Indeed, we would conclude saying that the most interesting finding of the current study is the increase of FCT after SGLT2-I therapy at 1 year of follow-up. Thus, we could consider SGLT2-I therapy similar effects on coronary plaques as compared to PCSK9 inhibitors. Notably, among patients with acute myocardial infarction, the addition of subcutaneous biweekly alirocumab to high-intensity statin therapy, compared with placebo, resulted in significantly greater coronary plaque regression in non-infarct-related arteries after 52 weeks [28]. Therefore, the combination of statin and evolocumab after an acute myocardial infarction produces favorable changes in coronary atherosclerosis, via the stabilization and regression of coronary plaque [29]. This therapy significantly reduces LDL-C levels in patients with acute coronary syndrome and consequently leads to the best clinical outcomes [29]. Furthermore, the significant lowering of LDL-C levels could be evidenced as a potential therapeutic mechanism for improved clinical outcomes in these patients [29]. In this scenario, we could hypothesize that the PCSK9i improve FCT by reducing LDL-C, while the SGLT2 probably increased FCT by reducing vascular inflammation and endothelial dysfunction.

The current study evidenced a few limitations. First, the patients were not randomized to the SGLT2-I therapy, which could result in a study bias. On the other hand, the SGLT2-I were not prescribed at the study beginning (2013) and were introduced later in clinical practice as oral anti-T2DM medication. Thus, this could cause the loss of randomization to SGLT-I therapy in the current study. On the other hand, this could represent a clear pic from current real-world clinical practice in the management of T2DM patients and in those with stable IHD and diagnosis of Mv-NOCS. Conversely, this is an observational (real world) study, which is prone to bias. Second, the study follow-up duration of 1 year could limit the generalizability of study results. Third, the number of enrolled patients could reduce the power of the current study and the statistical significance reached for the primary and secondary endpoints of study. Fourth, we cannot identify a clear cut-off value to stage the stable vs. unstable coronary atherosclerotic plaque phenotype in the current study and/or to predict its rupture with the consequent adverse clinical event. On the other hand, this study limitation is under other recent studies conducted on plaque morphology and thickness via OCT evaluation [28–31]. Finally, but not less relevant, in the current study, we found a significant reduction of MACEs at 1 year of follow-up in the SGLT2-I users vs. Non-SGLT2-I users. This result could confirm an ameliorative effect (MACEs reduction), induced by SGLT2-I at 1 year of follow-up. In our study, the MACEs represent a composite endpoint, containing distinct component endpoints, both coronary and non-coronary events, and including soft clinical endpoints. To date, the MACEs endpoint analysis and interpretation could be challenging, and evidence to the potential for widespread distribution of misleading study results [32]. Moreover, this could be evidenced as the result of an explorative analysis, because the small simple size and short duration of follow-up could represent a limit for this study result. Therefore, we could report that the causality between SGLT2-I use, and future adverse cardiovascular event cannot be concluded by the present study. Furthermore, these effects SGLT2-I induced on FCT and MACEs will be evaluated in a future larger study at a longer follow-up duration.

## Conclusions

In our study, SGLT2-I reduced by half the MACEs in the Mv-NOCS diabetic patients (best clinical outcomes). The introduction of SGLT2-I therapy post coronary angiography and OCT may be predictive of the 65% of risk reduction of MACEs at 1 year of follow-up. According to our data, this could result by the best glucose homeostasis, and reduction of systemic inflammatory burden, such as the local ameliorative effects on atherosclerotic plaque lipids’ deposit, inflammation and thickness, on coronary plaque in Mv-NOCS patients with T2DM.

## Data Availability

Not applicable.

## Abbreviations

SGLT2-I: Sodium–glucose cotransporter 2 inhibitors
T2DM: Type 2 diabetes mellitus
MACEs: Major adverse cardiovascular events
IHD: Ischemic heart disease
Mv: Multi vessel
NOCS: Non-obstructive coronary artery lesions
FCT: Fibrous cap thickness
OCT: Optical coherence tomography
FFR: Fractional flow reserve
PCI: Percutaneous coronary intervention
NLRP3: NLR family pyrin domain containing 3 inflammasome
IL1β: Interleukin 1 beta
NITs: Non-invasive stress test
TC: Total cholesterol
HDL-C: High density lipoprotein cholesterol
LDL-C: Low density lipoprotein cholesterol
BNP: B type natriuretic peptide
LMT: Left main trunk
LAD: Left anterior descending
LCX: Left circumflex
RCA: Right coronary artery
CFR: Coronary flow reserve
IVUS: Intravascular ultrasound
T0: Baseline
T1: After 24 h
T2: 6 Months
T3: 12 Months
Hb1Ac: Glycated hemoglobin
hs-TnT: High sensitivity T troponin
BMI: Body mass index
WBC: White blood cells
CRP: C reactive protein
IL-6: Interleukin-6
TNFα: Tumor necrosis factor alpha

## References

[1] Heerspink HJ, Perkins BA, Fitchett DH, Husain M, Cherney DZ (2016). Sodium glucose cotransporter 2 inhibitors in the treatment of diabetes mellitus: cardiovascular and kidney effects, potential mechanisms, and clinical applications. Circulation.

[2] Sardu C, Massetti M, Testa N, Martino LD, Castellano G, Turriziani F, Sasso FC, Torella M, De Feo M, Santulli G, Paolisso G, Marfella R (2021). Effects of sodium–glucose transporter 2 inhibitors (SGLT2-I) in patients with ischemic heart disease (IHD) treated by coronary artery bypass grafting *via* MiECC: inflammatory burden, and clinical outcomes at 5 years of follow-up. Front Pharmacol.

[3] Paolisso P, Bergamaschi L, Santulli G, Gallinoro E, Cesaro A, Gragnano F, Sardu C, Mileva N, Foà A, Armillotta M, Sansonetti A, Amicone S, Impellizzeri A, Casella G, Mauro C, Vassilev D, Marfella R, Calabrò P, Barbato E, Pizzi C (2022). Infarct size, inflammatory burden, and admission hyperglycemia in diabetic patients with acute myocardial infarction treated with SGLT2-inhibitors: a multicenter international registry. Cardiovasc Diabetol.

[4] Marfella R, Sardu C, Balestrieri ML, Siniscalchi M, Minicucci F, Signoriello G, Calabrò P, Mauro C, Pieretti G, Coppola A, Nicoletti G, Rizzo MR, Paolisso G, Barbieri M (2018). Effects of incretin treatment on cardiovascular outcomes in diabetic STEMI-patients with culprit obstructive and multivessel non obstructive-coronary-stenosis. Diabetol Metab Syndr.

[5] Milzi A, Burgmaier M, Burgmaier K (2017). Type 2 diabetes mellitus is associated with a lower fibrous cap thickness but has no impact on calcification morphology: an intracoronary optical coherence tomography study. Cardiovasc Diabetol.

[6] Sardu C, D'Onofrio N, Torella M, Portoghese M, Mureddu S, Loreni F, Ferraraccio F, Panarese I, Trotta MC, Gatta G, Galdiero M, Sasso FC, D'Amico M, De Feo M, Balestrieri ML, Paolisso G, Marfella R (2021). Metformin therapy effects on the expression of sodium–glucose cotransporter 2, leptin, and SIRT6 levels in pericoronary fat excised from pre-diabetic patients with acute myocardial infarction. Biomedicines.

[7] D'Onofrio N, Sardu C, Trotta MC, Scisciola L, Turriziani F, Ferraraccio F, Panarese I, Petrella L, Fanelli M, Modugno P, Massetti M, Marfella LV, Sasso FC, Rizzo MR, Barbieri M, Furbatto F, Minicucci F, Mauro C, Federici M, Balestrieri ML, Paolisso G, Marfella R (2021). Sodium–glucose co-transporter2 expression and inflammatory activity in diabetic atherosclerotic plaques: effects of sodium–glucose co-transporter2 inhibitor treatment. Mol Metab.

[8] Kim SR, Lee SG, Kim SH (2020). SGLT2 inhibition modulates NLRP3 inflammasome activity via ketones and insulin in diabetes with cardiovascular disease. Nat Commun.

[9] Sánchez-García A, Simental-Mendía M, Millán-Alanís JM, Simental-Mendía LE (2020). Effect of sodium–glucose co-transporter 2 inhibitors on lipid profile: a systematic review and meta-analysis of 48 randomized controlled trials. Pharmacol Res.

[10] Szekeres Z, Toth K, Szabados E (2021). The effects of sglt2 inhibitors on lipid metabolism. Metabolites.

[11] Scicali R, Giral P, D'Erasmo L, Cluzel P, Redheuil A, Di Pino A, Rabuazzo AM, Piro S, Arca M, Béliard S, Purrello F, Bruckert E, Gallo A (2021). High TG to HDL ratio plays a significant role on atherosclerosis extension in prediabetes and newly diagnosed type 2 diabetes subjects. Diabetes Metab Res Rev.

[12] Moghissi ES, Korytkowski MT, DiNardo M, American Association of Clinical Endocrinologists, American Diabetes Association (2009). American Association of Clinical Endocrinologists and American Diabetes Association consensus statement on inpatient glycemic control. Diabetes Care.

[13] Joseph JJ, Deedwania P, Acharya T, Aguilar D, Bhatt DL, Chyun DA, Di Palo KE, Golden SH, Sperling LS, American Heart Association Diabetes Committee of the Council on Lifestyle and Cardiometabolic Health, Council on Arteriosclerosis, Thrombosis and Vascular Biology, Council on Clinical Cardiology, Council on Hypertension (2022). Comprehensive management of cardiovascular risk factors for adults with type 2 diabetes: a scientific statement from the American Heart Association. Circulation.

[14] Bashier A, Bin Hussain A, Abdelgadir E (2019). Consensus recommendations for management of patients with type 2 diabetes mellitus and cardiovascular diseases. Diabetol Metab Syndr.

[15] Xing L, Higuma T, Wang Z, Aguirre AD, Mizuno K, Takano M, Dauerman HL, Park SJ, Jang Y, Kim CJ, Kim SJ, Choi SY, Itoh T, Uemura S, Lowe H, Walters DL, Barlis P, Lee S, Lerman A, Toma C, Tan JWC, Yamamoto E, Bryniarski K, Dai J, Zanchin T, Zhang S, Yu B, Lee H, Fujimoto J, Fuster V, Jang IK (2017). Clinical significance of lipid-rich plaque detected by optical coherence tomography: a 4-year follow-up study. J Am Coll Cardiol.

[16] Tearney GJ, Regar E, Akasaka T (2012). Consensus standards for acquisition, measurement, and reporting of intravascular optical coherence tomography studies: a report from the international working group for intravascular optical coherence tomography standardization and validation. J Am Coll Cardiol.

[17] Sardu C, Paolisso P, Sacra C, Mauro C, Minicucci F, Portoghese M, Rizzo MR, Barbieri M, Sasso FC, D'Onofrio N, Balestrieri ML, Calabrò P, Paolisso G, Marfella R (2019). Effects of metformin therapy on coronary endothelial dysfunction in patients with prediabetes with stable angina and nonobstructive coronary artery stenosis: the CODYCE multicenter prospective study. Diabetes Care.

[18] Sardu C, Gatta G, Pieretti G, Viola L, Sacra C, Di Grezia G, Musto L, Minelli S, La Forgia D, Capodieci M, Galiano A, Vestito A, De Lisio A, Pafundi PC, Sasso FC, Cappabianca S, Nicoletti G, Paolisso G, Marfella R (2021). Pre-menopausal breast fat density might predict MACE during 10 years of follow-up: the BRECARD study. JACC Cardiovasc Imaging.

[19] Windecker S, Kolh P, Alfonso F, Collet JP, Cremer J, Falk V, Filippatos G, Hamm C, Head SJ, Authors/Task Force m (2014). ESC/EACTS guidelines on myocardial revascularization: the Task Force on myocardial revascularization of the European Society of Cardiology (ESC) and the European Association for Cardio-Thoracic Surgery (EACTS) developed with the special contribution of the European Association of Percutaneous Cardiovascular Interventions (EAPCI). Eur Heart J.

[20] Shaw LJ, Berman DS, Picard MH, Friedrich MG, Kwong RY, Stone GW, Senior R, Min JK, Hachamovitch R, Scherrer-Crosbie M (2014). 27. Comparative definitions for moderate-severe ischemia in stress nuclear, echocardiography, and magnetic resonance imaging. JACC Cardiovasc Imaging.

[21] Kunadian V, Chieffo A, Camici PG, Berry C, Escaned J, Maas A, Prescott E, Karam N, Appelman Y, Fraccaro C (2020). An EAPCI expert consensus document on ischaemia with non-obstructive coronary arteries in collaboration with European Society of Cardiology Working Group on Coronary Pathophysiology & microcirculation endorsed by coronary vasomotor disorders international study group. Eur Heart J.

[22] Barlis P, Serruys PW, Gonzalo N (2008). Assessment of culprit and remote coronary narrowings using optical coherence tomography with long-term outcomes. Am J Cardiol.

[23] Mitchell C, Rahko PS, Blauwet LA, Canaday B, Finstuen JA, Foster MC, Horton K, Ogunyankin KO, Palma RA, Velazquez EJ (2019). Guidelines for performing a comprehensive transthoracic echocardiographic examination in adults: recommendations from the American Society of Echocardiography. J Am Soc Echocardiogr.

[24] Oshima H, Miki T, Kuno A, Mizuno M, Sato T, Tanno M, Yano T, Nakata K, Kimura Y, Abe K, Ohwada W, Miura T (2019). Empagliflozin, an SGLT2 inhibitor, reduced the mortality rate after acute myocardial infarction with modification of cardiac metabolomes and antioxidants in diabetic rats. J Pharmacol Exp Ther.

[25] Xiang B, Zhao X, Zhou X (2021). Cardiovascular benefits of sodium–glucose cotransporter 2 inhibitors in diabetic and nondiabetic patients. Cardiovasc Diabetol.

[26] Marfella R, D'Onofrio N, Trotta MC, Sardu C, Scisciola L, Amarelli C, Balestrieri ML, Grimaldi V, Mansueto G, Esposito S, D'Amico M, Golino P, Signoriello G, De Feo M, Maiello C, Napoli C, Paolisso G (2022). Sodium/glucose cotransporter 2 (SGLT2) inhibitors improve cardiac function by reducing JunD expression in human diabetic hearts. Metabolism.

[27] Sardu C, Massimo Massetti M, Rambaldi P, Gatta G, Cappabianca S, Sasso FC, Santamaria M, Volpicelli M, Ducceschi V, Signoriello G, Paolisso G, Marfella R (2022). SGLT2-inhibitors reduce the cardiac autonomic neuropathy dysfunction and vaso-vagal syncope recurrence in patients with type 2 diabetes mellitus: the SCAN study. Metabolism.

[28] Räber L, Ueki Y, Otsuka T, Losdat S, Häner JD, Lonborg J, Fahrni G, Iglesias JF, van Geuns RJ, Ondracek AS, Radu Juul Jensen MD, Zanchin C, Stortecky S, Spirk D, Siontis GCM, Saleh L, Matter CM, Daemen J, Mach F, Heg D, Windecker S, Engstrøm T, Lang IM, Koskinas KC, PACMAN-AMI collaborators (2022). Effect of alirocumab added to high-intensity statin therapy on coronary atherosclerosis in patients with acute myocardial infarction: the PACMAN-AMI randomized clinical trial. JAMA.

[29] Nicholls SJ, Kataoka Y, Nissen SE, Prati F, Windecker S, Puri R, Hucko T, Aradi D, Herrman JR, Hermanides RS, Wang B, Wang H, Butters J, Di Giovanni G, Jones S, Pompili G, Psaltis PJ (2022). Effect of evolocumab on coronary plaque phenotype and burden in statin-treated patients following myocardial infarction. JACC Cardiovasc Imaging.

[30] Libby P (2013). Mechanisms of acute coronary syndromes and their implications for therapy. N Engl J Med.

[31] Kedhi E, Berta B, Roleder T, Hermanides RS, Fabris E, Ijsselmuiden AJJ, Kauer F, Alfonso F, von Birgelen C, Escaned J, Camaro C, Kennedy MW, Pereira B, Magro M, Nef H, Reith S, Al Nooryani A, Rivero F, Malinowski K, De Luca G, GarciaGarcia H, Granada JF, Wojakowski W (2021). Thin-cap fibroatheroma predicts clinical events in diabetic patients with normal fractional flow reserve: the COMBINE OCT-FFR trial. Eur Heart J.

[32] Lauer MS, Topol EJ (2003). Clinical trials—multiple treatments, multiple end points, and multiple lessons. JAMA.

